# Conceptual design of a high-speed electromagnetic switch for a modified flux-coupling-type SFCL and its application in renewable energy system

**DOI:** 10.1186/s40064-016-2347-6

**Published:** 2016-06-17

**Authors:** Lei Chen, Hongkun Chen, Jun Yang, Zhengyu Shu, Huiwen He, Xin Shu

**Affiliations:** School of Electrical Engineering, Wuhan University, NO. 8, Donghu South Road, Wuchang District, Wuhan, 430072 Hubei China; State Grid Yichang Power Supply Company, Yichang, 443000 China; China Electric Power Research Institute, Wuhan, 430074 China; State Grid Hubei Electric Power Research Institute, Wuhan, 430077 China

**Keywords:** Conceptual design, Electromagnetic repulsion mechanism (ERM), Flux-coupling-type SFCL, High-speed switch, Micro-grid system

## Abstract

The modified flux-coupling-type superconducting fault current (SFCL) is a high-efficient electrical auxiliary device, whose basic function is to suppress the short-circuit current by controlling the magnetic path through a high-speed switch. In this paper, the high-speed switch is based on electromagnetic repulsion mechanism, and its conceptual design is carried out to promote the application of the modified SFCL. Regarding that the switch which is consisting of a mobile copper disc, two fixed opening and closing coils, the computational method for the electromagnetic force is discussed, and also the dynamic mathematical model including circuit equation, magnetic field equation as well as mechanical motion equation is theoretically deduced. According to the mathematical modeling and calculation of characteristic parameters, a feasible design scheme is presented, and the high-speed switch’s response time can be less than 0.5 ms. For that the modified SFCL is equipped with this high-speed switch, the SFCL’s application in a 10 kV micro-grid system with multiple renewable energy sources are assessed in the MATLAB software. The simulations are well able to affirm the SFCL’s performance behaviors.

## Background

For the modern electric power systems, one of the major challenges and demands is how to solve the continually increasing fault currents (Shuai et al. 2015; Li et al. 2015). In the case of that the fault currents cannot be timely limited and interrupted, a number of problems will be caused, such as the failure of electric device and the expansion of power supply interrupted region (Lapthorn et al. 2011; Im et al. 2014). At present, owing to the unique technical advantages of superconducting materials (Malginov et al. 2013a, b; Guan et al. 2015), superconducting fault current limiters (SFCLs) can be thought of as the highly competitive auxiliary devices to assist the electric power systems against short-circuit faults. On this background, lots of research works have been carried out, and different kinds of SFCLs have been proposed (Didier and Lévêque 2014; Firouzi et al. 2015; Chen et al. 2015a, b; Martini et al. 2015; Reiss 2015).

In terms of the proposed SFCLs, they can be approximately classified as resistive and inductive SFCLs. Our research group has suggested a modified flux-coupling-type SFCL, which is a resistive-inductive type (hybrid type) SFCL, and is able to theoretically enhance the transient performance of an electrical system more efficiently (Chen et al. 2015c, d; Deng et al. 2015). This flux-coupling-type SFCL is to suppress the short-circuit current by controlling the magnetic path through a high-speed switch. In other words, the high-speed switch’s operating characteristics will directly affect the SFCL’s performance behaviors and engineering application. From this perspective, the study of a feasible design scheme for the switch is necessary and meaningful.

In this paper, the conceptual design of the high-speed switch with electromagnetic repulsion mechanism (ERM) used in a 10 kV class SFCL is done, and also the application of the SFCL in a micro-grid system with multiple renewable energy sources is assessed. The ‎article is organized in the following manner. “Theoretical analysis” section presents the SFCL’s structural principle, discusses the computational method for the high-speed switch’s electromagnetic force, and builds the dynamic mathematical model. In “Numerical calculation and simulation study” section, calculation analyses and transient simulations are performed to verify the electromagnetic switch’s design scheme and the SFCL’s performance. In “Conclusions” section, conclusions are summarized and next steps are prospected.

## Theoretical analysis

### Theoretical presentation of the flux-coupling-type SFCL

**Fig. 1:**
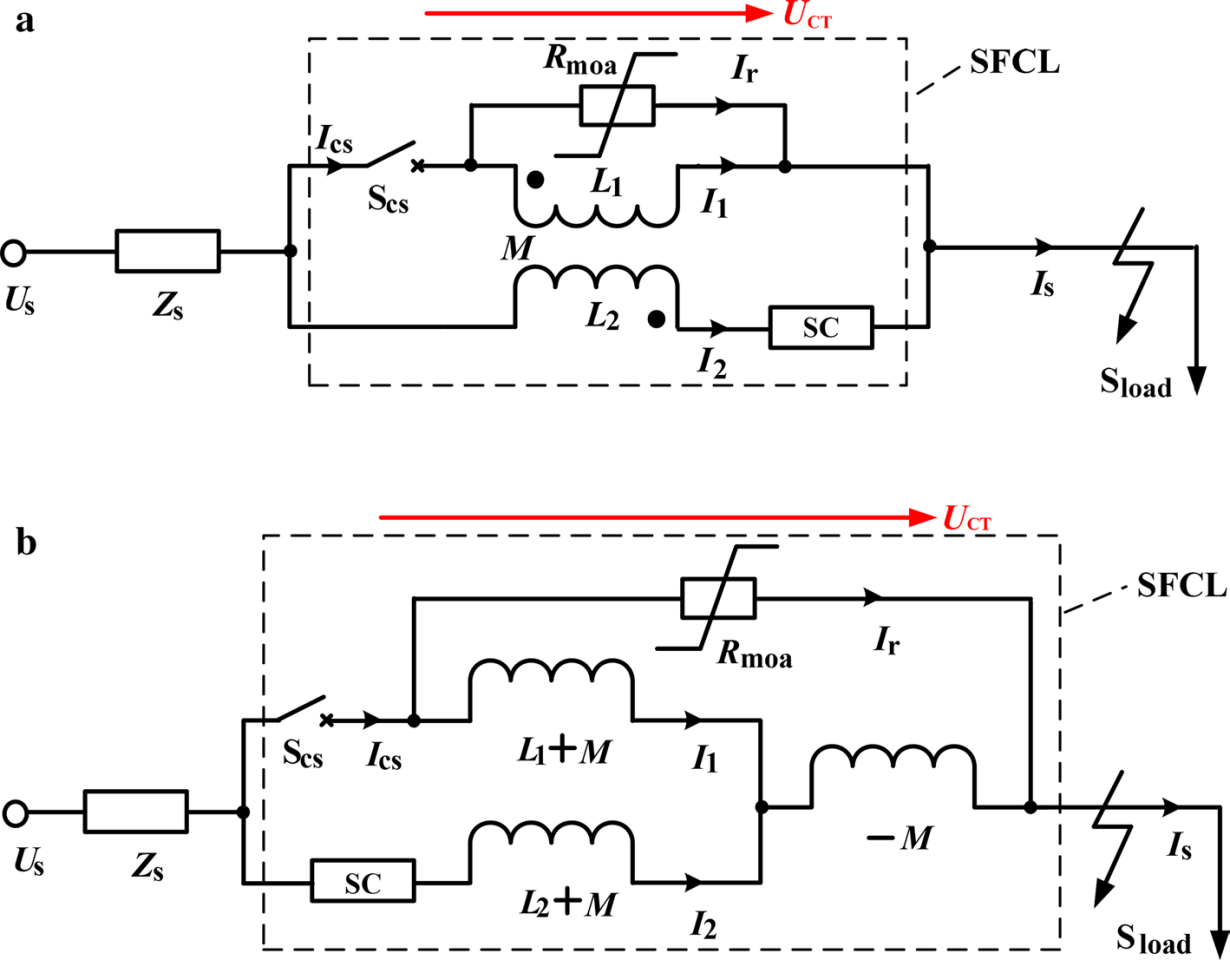
Configuration structure of a modified flux-coupling-type SFCL. **a** Main connection and **b** electrical equivalent circuit

The schematic diagram of the flux-coupling-type SFCL is shown in Fig. 1a. This SFCL is mainly consisting of a coupling transformer (CT), a controlled high-speed switch S_cs_ and a superconducting coil (SC). The switch S_cs_ and the SC are respectively connected in series with the CT’s primary and secondary windings, which are wound in reverse directions. The metal oxide arrester (MOA), whose expected function is to suppress switching overvoltage, is connected in parallel with the CT. *L*_1_, *L*_2_ are recorded as the winding self-inductances, respectively, and *M* is the mutual inductance. In addition, *Z*_s_ is the circuit impedance and *S*_load_ is the circuit load. *R*_SC_/*R*_moa_ is recorded as the SC/MOA’s normal-state resistance.

Since it is convenient to investigate the CT’s characteristics by using the equivalent circuit whose parameters are expressed in terms of mutual-inductance and self-inductances, the SFCL’s impedance characteristic can be studied more clearly. According to the CT’s equivalent circuit, the SFCL’s electrical equivalent structure is shown in Fig. 1b.

In normal condition, S_cs_ is under the closed state and the SC is maintained in the zero-resistance state. Herein, the CT’s operating impedance *Z*_ct_ will play a decisive role on the SFCL’s performance characteristic. The analysis of the impedance *Z*_CT_ is conducted. Actually, *Z*_CT_ is the ratio of the transformer voltage *U*_CT_ and the transformer current *I*_CT_. The voltage *U*_CT_ denotes the voltage over the two terminals of the CT’s primary or secondary inductance, and it can be shown in Fig. 1. In regard to the current *I*_CT_, it denotes the total currents which respectively flow through the CT’s primary inductance *L*_1_ and secondary inductance *L*_2_. And namely if the CT can be regarded as a black box with an input current and a same output current, *I*_CT_ is the sum of *I*_1_ and *I*_2_. From the electric relationship demonstrated in Fig. 1, *Z*_CT_ can be expressed as:

1 $$\begin{aligned} Z_{CT} = \frac{{U_{CT} }}{{I_{CT} }} &= \text{j} {{\upomega }}\left[\frac{{(L_{1} + M)(L_{2} + M)}}{{(L_{1} + M) + (L_{2} + M)}} - M \right]\\ &= \text{j} {{\upomega }}\frac{{L_{1} L_{2} - M^{2} }}{{L_{1} + L_{2} + 2M}} \hfill \\ \end{aligned}$$

When the coupling coefficient *k* and the transformation ratio *n* can be respectively expressed as $k = M/\sqrt {L_{1} L_{2} }$ and $n = \sqrt {L_{1} /L_{2} }$, $Z_{CT} = \varvec{j}{{\upomega }}L_{2} (1 - k^{2} )n^{2} /(n^{2} + 2kn + 1)$ is obtained. In the case of that an iron core is used to maximize the coupling, *k* will be approximate to 1 and *Z*_CT_ ≈ 0. The non-inductive coupling is achieved, and the MOA is “short-circuited”. Consequently, the SFCL will not affect the main circuit.

After the short-circuit fault happens, S_cs_ will be opened rapidly, and meanwhile the MOA may suppress the overvoltage caused by the switching operation. Since the electromagnetic relationship is changed by the controlled switch, the non-inductive coupling will pass away, and also the fault current in the SC will make the superconductor be quenching. The modified SFCL’s current-limiting impedance can be calculated as:

2 $$\begin{aligned} Z_{SFCL}& = [{\mathbf{I}}_{{\mathbf{2}}}^{{\mathbf{'}}} \varvec{(}R_{\text{SC}} + \varvec{j}{{\upomega }}L_{2} ) - {\mathbf{I}}_{{\mathbf{1}}}^{{\mathbf{'}}} \varvec{j}{{\upomega }}M]/{\mathbf{I}}_{{\mathbf{2}}}^{{\mathbf{'}}} \\ &= R_{\text{SC}} + \varvec{j}{{\upomega }}L_{2} - {\mathbf{I}}_{{\mathbf{1}}}^{{\mathbf{'}}} \varvec{j}{{\upomega }}M/{\mathbf{I}}_{{\mathbf{2}}}^{{\mathbf{'}}} \\ \end{aligned}$$

Where ${\mathbf{I}}_{{\mathbf{1}}}^{{\mathbf{'}}}$ and ${\mathbf{I}}_{{\mathbf{2}}}^{{\mathbf{'}}}$ denote the steady-state vector-currents flowing through the CT’s primary and secondary inductances. As the controlled switch S_cs_ has been opened, the physical-circuit electrical connection between the CT’s primary and secondary inductances will be replaced by the magnetic-circuit coupling connection. According to the inductive coupling characteristic (Chen et al. 2010, 2014), the relationship between the two currents can be derived by:

3 $$\begin{aligned} &\varvec{j}{{\upomega L}}_{1} {\mathbf{I}}_{{\mathbf{1}}}^{{\mathbf{'}}} - \varvec{j}{{\upomega }}M{\mathbf{I}}_{{\mathbf{2}}}^{{\mathbf{'}}} = - {\mathbf{I}}_{{\mathbf{1}}}^{{\mathbf{'}}} {\text{R}}_{\text{moa}} \hfill \\ &\Rightarrow {\mathbf{I}}_{{\mathbf{1}}}^{{\mathbf{'}}} \varvec{(j}{{\upomega L}}_{1} + {\text{R}}_{\text{moa}} ) = \varvec{j}{{\upomega }}M{\mathbf{I}}_{{\mathbf{2}}}^{{\mathbf{'}}} \hfill \\ &\Rightarrow \frac{{{\mathbf{I}}_{{\mathbf{1}}}^{{\mathbf{'}}} }}{{{\mathbf{I}}_{{\mathbf{2}}}^{{\mathbf{'}}} }} = \frac{{\varvec{j}{{\upomega }}M}}{{\varvec{j}{{\upomega L}}_{1} {\text{ + R}}_{\text{moa}} }} \hfill \\ \end{aligned}$$

According to Eqs. (2) and (3), the impedance *Z*_SFCL_ can be rewritten as: $Z_{SFCL} = [R_{\text{SC}} + {\text{j}}\omega L_{ 2} + \left( {kn\omega L_{ 2} } \right)^{ 2} /\left( {R_{moa} + n^{ 2} \omega L_{ 2} } \right)$. In view of $R_{moa} \gg n^{ 2} \omega L_{ 2} ,Z_{SFCL} \approx R_{\text{SC}} + {\text{j}}\omega L_{ 2}$ can be obtained. Compared to the original flux-coupling-type SFCL which is purely inductive (Ren et al. 2010; Chen et al. 2015e), the suggested SFCL is a resistive-inductive type (hybrid type) SFCL, which can potentially bring more contributions, such as inhibiting the fluctuations of active power and reactive power, restraining electromagnetic oscillations as well as providing critical protection to relevant power equipment (Chen et al. 2009).

In light of the SFCL’s structure and principle, the following requirements related to the high-speed switch can be obtained: (1) the switch may accurately interrupt the coil current to ensure the SFCL’s reliable action; (2) the switch may carry out the interrupting operation within milliseconds to ensure the SFCL’s rapid response. Considering the practical application of switch technology in the field of SFCL, a high-speed switch based on electromagnetic repulsion mechanism (ERM) has received lots of attention from research scholars (Lim et al. 2010, 2014; He and Wang 2014). Herein this kind of switch is adopted for the modified flux-coupling-type SFCL, and the switch’s operating principle and mathematical modeling will be stated in the next section.

### Principle and modeling of the switch with ERM

**Fig. 2:**
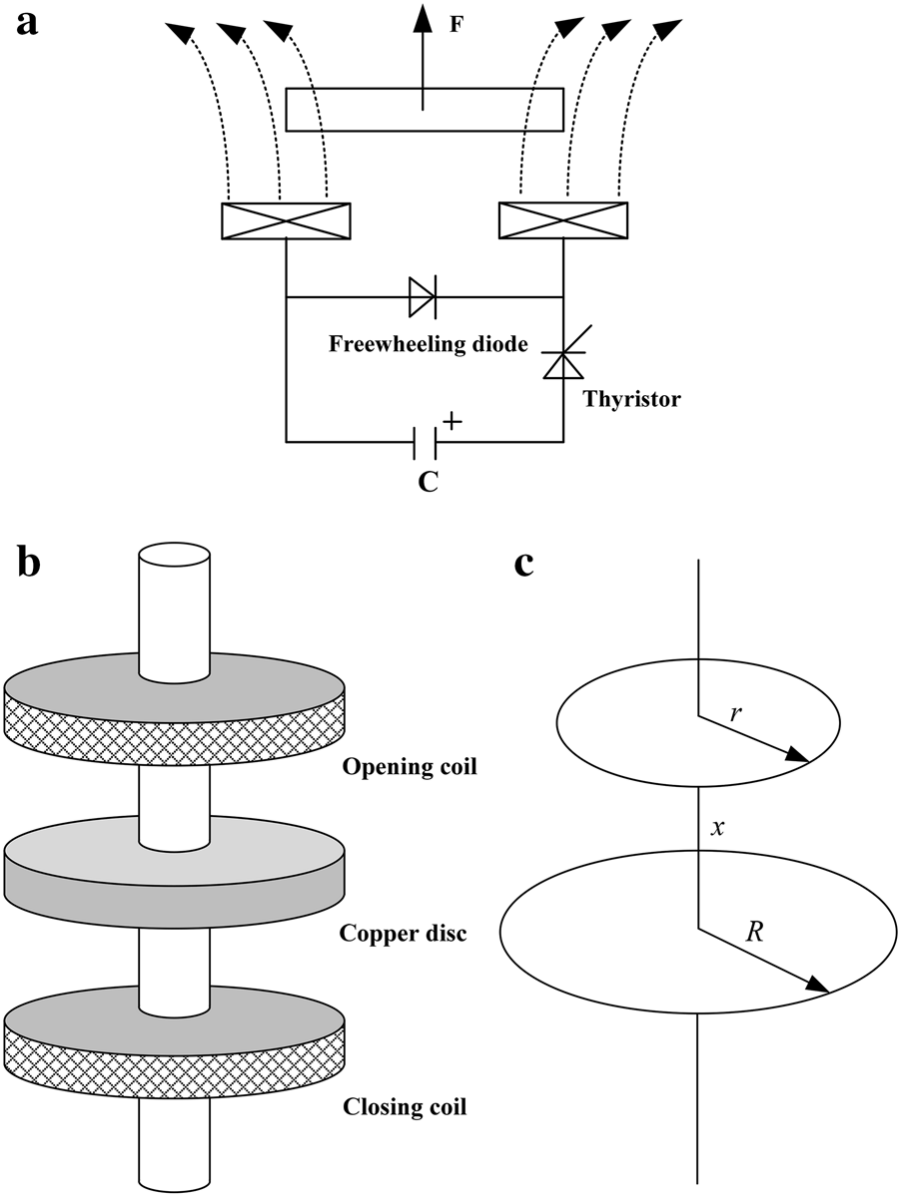
High-speed electromagnetic switch with ERM. **a** Working circuit, **b** spatial topology structure and **c** simplified schematic diagram for characteristic analysis

Figure 2a shows the working circuit of electromagnetic repulsion mechanism (ERM), and Fig. 2b indicates the spatial topology structure of the selected switch with ERM. This switch is mainly consisting of a mobile copper disc, two fixed opening and closing coils. When the opening or closing coil is electrified by a presupposed capacitor, eddy current will be induced in the copper disc. Further, the electromagnetic repulsion force will appear to drive the copper disc’s link system, and the open or close operation can be done. To compute the electromagnetic force, a finite element method (FEM) is usually adopted (Ranlöf et al. 2013; Peng et al. 2014; Najafi and Iskender 2016). Considering that although FEM can analyze magnetic field and eddy current more intuitively, sometimes its modeling and calculation procedures are relatively complicated. Instead of it, a mathematical calculation method is applied.

For either the mobile copper disc or the electrified opening/closing coil, it can be equivalent to multiple coaxial turns of wire-loops with different radiuses (Li et al. 2011). Thus, regarding the basic computational unit for analyzing the electromagnetic interaction between the copper disc and the electrified coil, it is equivalent to any two coaxial single-turn coils, as shown in Fig. 2c. Herein *r* and *R* are respectively the two single-turn coils’ radiuses, and *x* is the spatial distance between them. According to electromagnetic theory, the mutual inductance *M*_st_ between the two coils can be expressed as:

4 $$M_{st} = \mu_{0} \sqrt {Rr} \left[ {({2/ { {k_{st} - k_{st} }} {k_{st} - k_{st} }})B(k_{st} ) - E(k_{st} ){2/ {k_{st} } }} \right]$$

where *k*_*st*_, B(*k*_*st*_), and *E*(*k*_*st*_) are respectively expressed as $k_{st} = 2\sqrt {\frac{Rr}{{x^{2} + (R + r)^{2} }}}$, $B(k_{st} ) = \int_{0}^{\pi /2} {\frac{d\alpha }{{\sqrt {1 - k_{st}^{2} \sin^{2} \alpha } }}}$, $E(k_{st} ) = \int_{0}^{\pi /2} {\sqrt {1 - k_{st}^{2} \sin^{2} \alpha } d\alpha }$.

Supposing that the currents flowing through the two single-turn coils can be respectively expressed as *i*_0_ and *i*_1_, the electromagnetic force *F*_st_ between them can be shown in:

5 $$\begin{aligned} F_{st} &= i_{0} i_{1} dM_{st} /x \\& = i_{0} i_{1} \frac{{2\mu_{0} xRr}}{{[x^{2} + (R + r)^{2} ]^{3/2} }}\int_{0}^{\pi /2} {\frac{\cos 2\alpha d\alpha }{{(1 - k_{st}^{2} \sin^{2} \alpha )^{3/2} }}} \end{aligned}$$

In a similar way, the total electromagnetic force *F*_total_ between the electrified coil and the copper disc is computed in:

6 $$\left\{ \begin{aligned} \text{ }F_{total} = \sum\nolimits_{j = 1}^{{N_{0} }} {\sum\nolimits_{p = 1}^{{N_{1} }} {i_{e} } } i_{p} \frac{{dM_{jp} }}{dx}\text{ } \hfill \\ m\frac{{d^{2} x}}{{dt^{2} }} = F_{total} - f(x) \hfill \\ \end{aligned} \right.$$

where *N*_0_ is the number of turns of the electrified coil; *i.*e. is the current flowing through the electrified coil; the copper disc is equivalent to *N*_1_ turns of wire-loops in which different eddy currents *i*_p_ are induced; *M*_jp_ is the mutual inductance between any two coaxial single-turn coils; *m* expresses the mass of the copper disc and its link system; *f* (*x*) denotes the movement resistance including gravity as well as friction, and the mechanical motion equation related to the spatial distance *x* is also given. For the calculation of different eddy currents (*i*_1_, *i*_2_, *i*_3_…*i*_*N1*_), Eq. (7) can be obtained.

7 $$\left[ {\begin{array}{*{20}c} {{\text{i}}_{e} } \\ {i_{1} } \\ {i_{2} } \\ \vdots \\ {i_{{N_{1} }} } \\ \end{array} } \right] = \left[ {\begin{array}{*{20}c} {\frac{{E_{e} - \frac{1}{{C_{e} }}\int\limits_{0}^{t} {i_{e} dt} }}{{\sum\nolimits_{j = 1}^{{N_{0} }} {R_{j} } }}} \\ 0 \\ 0 \\ \vdots \\ 0 \\ \end{array} } \right] - \left[ {\mathbf{A}} \right]\left[ {\begin{array}{*{20}c} {\frac{{{\text{di}}_{e} }}{dt}} \\ {\frac{{{\text{di}}_{1} }}{dt}} \\ {\frac{{{\text{di}}_{2} }}{dt}} \\ \vdots \\ {\frac{{{\text{di}}_{{N_{1} }} }}{dt}} \\ \end{array} } \right]$$

where *C*_*e*_ is the charging capacitance; *E*_*e*_ is the capacitor voltage’s initial value; *R*_j_ indicates a single-turn coil’s electrical resistance; **A** is the inductance coefficient matix with *N*_1_ + 1 dimension (Li et al. 2004).

In regard to Eq. (7), its expression by using vector–matrix can be written as:

8 $${\mathbf{I}} = {\mathbf{E}} - {\mathbf{A}}\frac{{d{\mathbf{I}}}}{dt}$$

9 $$\frac{{d{\mathbf{I}}}}{dt} = {\mathbf{A}}^{ - 1} ({\mathbf{E}} - {\mathbf{I}})$$

Through the discretization process, the recurrence equation of the currents under time domain can be derived as:

10 $$\left\{ \begin{array}{l} {\mathbf{I}}_{j + 1} = {\mathbf{A}}^{ - 1} ({\mathbf{E}}_{j + 1} - {\mathbf{I}}_{j + 1} )\Updelta t + {\mathbf{I}}_{j} \hfill \\ {\mathbf{I}}_{0} = 0 \hfill \\ \end{array} \right.$$

where Δ*t* is the calculation step; *j* is the discretization time.

On the basis of the aforementioned equations, theoretical analysis and mathematical calculation of the electromagnetic repulsion force can be performed, and further it can lay a foundation for the subsequent conceptual design of the switch.

## Numerical calculation and simulation study

### Design of the switch and its performance calculation

As the switch’s operating characteristic is determined by its electromagnetic force in principle, a few key factors related to the electromagnetic force are assessed during the numerical calculations. The initial conditions are set as *N*_0_ = 15, *N*_1_ = 20, *x* = 10 mm (maximum stroke), *r* = 15 mm, *R* = 12 mm, *E*_e_ = 200 V, Δ*t* = 1 μs, and *C*_e_ = 53,000 μF.

**Fig. 3:**
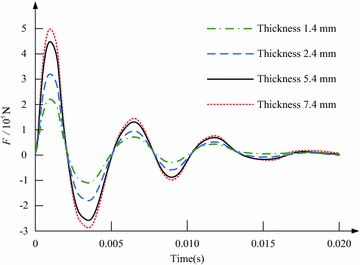
Influence of changing the copper disc’s thickness on the switch’s electromagnetic force

Figure 3 shows the influence of the copper disc’s thickness on the electromagnetic force. When the thickness is respectively set as 1.4, 2.4, 5.4 and 7.4 mm, the electromagnetic force’s peak value will reach to 232550N, 324820N, 458930N and 501390N, respectively. Corresponding to that, the switch’s response time is 0.96, 0.88, 0.8 and 0.78 ms, respectively. In spite of that increasing the thickness can lead to the rise of the electromagnetic force, this rising has a slower trend. It is not recommended to increase the thickness excessively, otherwise the skin effect will become obvious and the loss will be enlarged (Xing et al. 2015).

**Fig. 4:**
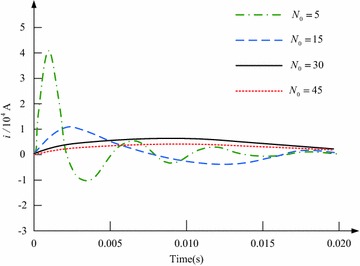
Waveform of the current across the electrified coil in the case that the number of turns of the coil is changed

**Fig. 5:**
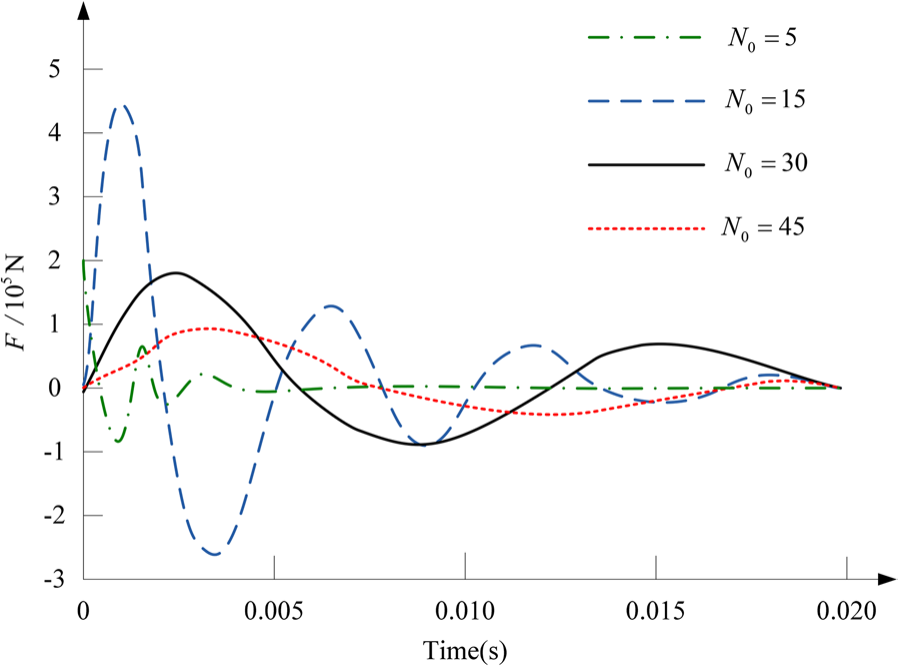
Influence of adjusting the number of turns of the electrified coil on the switch’s electromagnetic force

Figure 4 indicates the current across the electrified coil, and herein the number of turns of the coil is changed. The ratio of *N*_1_ to *N*_0_ is fixed to 1.3, and the copper disc’s thickness is set as 5.4 mm. It is observed that, the peak value of the current across the electrified coil will decrease with the increase of *N*_0_, and meanwhile the current’s rate of rise will be limited. Considering that the electromagnetic force has a close relation to the coil current, which is affected by the number of turns of the coil, Fig. 5 shows the impacts of adjusting the number of turns of the electrified coil on the switch’s electromagnetic force. The ratio of *N*_1_ to *N*_0_ is still fixed to 1.3, and the copper disc’s thickness is 5.4 mm. With the augment of *N*_0_, the electromagnetic force’s peak value will increase firstly and then decrease. For that *N*_0_ is respectively set as 5, 15, 30 and 45, the electromagnetic force’s peak value will reach to its maximum 458930N (*N*_0_ = 15) and minimum 91540N (*N*_0_ = 45), and the switch’s least and longest response time is respectively 0.8 and 2.56 ms.

**Fig. 6:**
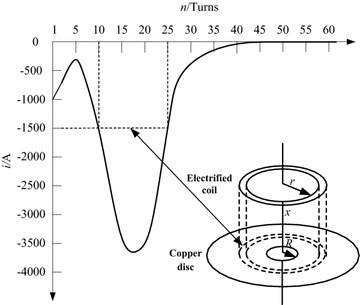
Peak-value characteristic of the eddy current induced in the copper disc’s each equivalent wire-loop (*N*_0_ = 15, *N*_1_ = 60)

To study how the relative size between the electrified coil and the copper disc can affect the switch’s electromagnetic force, Fig. 6 shows the peak value of the eddy current induced in the copper disc’s each equivalent wire-loop (the initial conditions are set as *N*_0_ = 15, *N*_1_ = 60, *x* = 10 mm, *r* = 15 mm, and *R* = 5 mm). Obviously, the eddy current appeared in the spatial overlap between the copper disc and the electrified coil is relatively large, and it will reach to the maximum in the copper disc’s 18th wire-loop. It may be inferred that, if the copper disc has the similar or comparable size as the electrified coil, the switch’s operating efficiency can be greatly improved. In view of it, this philosophy should be seriously carried out in the next design scheme.

**Fig. 7:**
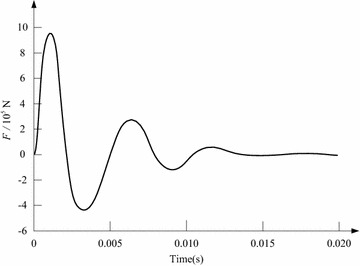
Electromagnetic force characteristic of the switch under the designed scheme

**Fig. 8:**
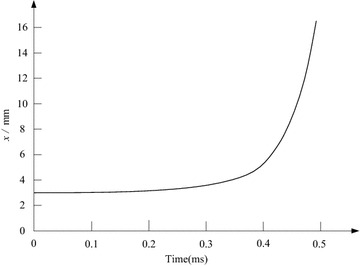
Operating process of the switch’s contact under the designed scheme

From the aforementioned numerical calculations, the design parameters of the switch are modified further, so as to adjust the structure, improve the electromagnetic force and reduce the response time. The design scheme is described as that *N*_0_ = 15, *N*_1_ = 25, *x* = 10 mm (contact maximum stroke), *r* = 30 mm, *R* = 20 mm, *E*_e_ = 220 V, *C*_e_ = 53000 μF, and the copper disc’s thickness is set as 5.4 mm. In accordance with this scheme, the electromagnetic force’s peak value can reach to 957640N, and the switch’s response time can be reduced to 0.45 ms. Figures 7 and 8 show the switch’s performance behaviors under the design scheme. Note that, during the following transient simulations, the SFCL will configure this high-speed electromagnetic switch, and the SFCL’s application in a micro-grid system with multiple renewable energy sources will be assessed in the MATLAB software.

### Application of the SFCL in a renewable energy system

**Fig. 9:**
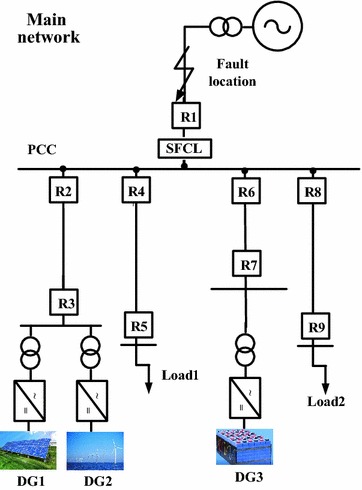
Application of the SFCL in a micro-grid system with DG units

As shown in Fig. 9, it indicates the application of the modified SFCL in a typical micro-grid system, which is composed of a PV generation (DG1), a wind plant (DG2), an energy storage device (DG3) as well as two loads. From this figure, all of the distributed generation (DG) units are connected to the micro-grid through the inverters, and this type of DG unit can be identified as the inverter interfaced distributed generation (IIDG). Note that, the main application objective of the SFCL is expected to improve the micro-grid’s robustness against the external fault, and the SFCL is installed at the point of common coupling (PCC) between the micro-grid and the main network. Moreover, a detailed transient model is built in MATLAB/Simulink, and the main simulation parameters are indicated in Table 1.

**Table 1:** Main simulation parameters of the system model

*Demonstrated micro-grid system*	
DG1/DG2/DG3	180 kW/100 kW/150 kW
Transmission line	0.27+j0.347 Ω/km, 2 km
Load 1/load 2	350 kW/250 kW
PCC voltage/frequency	10 kV/50 Hz
*Modified flux-coupling-type SFCL*	
Primary inductance	50 mH/70 mH
Coupling coefficient/coil ratio	0.99/0.5
Superconducting coil *R*_SC_	10 Ω/30 Ω

Concerning the dynamic simulation model of the DG1, its main circuit is described as follows. The PV array is connected to the input side of a boost converter and its output side is connected to the DC link capacitor. Further, a three-phase voltage source (VSI) is adopted to maintain constant DC voltage and supply sinusoidal current to the micro-grid. For the boost converter, the maximum power point tracking (MPPT) control is used to ensure the PV generation system’s operating efficiency. Details can be achieved in (Nanou and Papathanassiou 2014; Mohanty et al. 2014).

In regard to the modeling of the DG2, it is based on fixed-speed wind turbine (FSWT). FSWT technically adopts the induction generator (IG) and is connected to the micro-grid through a transformer. The MATLAB software library provides a standard model for the induction generator, and for the models of the wind turbine and the drivetrain system, details can be obtained in (Firouzi and Gharehpetian 2013; Ouchbel et al. 2014).

**Fig. 10:**
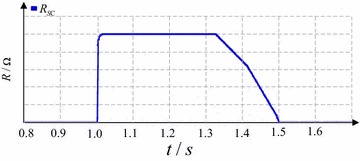
Quench/recovery model of the superconducting coil used in the SFCL

Considering the simulation modeling of the modified SFCL, the quench/recovery model of the superconducting coil is according to Fig. 10 (Moon et al. 2011). The SC’s transient characteristic can be expressed as:

11 $$R(t) = \left\{ {\begin{array}{*{20}ll} 0 &\quad {(t < t_{0} )} \\ {R_{n} \left[ {1 - \exp \left( { - \frac{{t - t_{0} }}{\tau }} \right)} \right]^{1/2} } & \quad{(t_{0} \le t < t_{1} )} \\ {a_{1} (t - t_{1} ) + b_{1} } & \quad{(t_{1} \le t < t_{2} )} \\ {a_{2} (t - t_{2} ) + b_{2} } &\quad {(t_{2} \le t < t_{3} )} \\ \end{array} } \right.$$

where *R*_n_ denotes the SFCL’s normal-state resistance; *τ* is the time constant. The SFCL’s time-domain characteristic is stated as that, *t*_0_, *t*_1_, and *t*_2_ indicate the quench-starting time, the first recovery-starting time, and the secondary recovery-starting time, respectively. *a*_1_, *b*_1_, *a*_2_, and *b*_2_ are respectively the function coefficients. During the simulation, it is designed that the SC will enter the normal state within 4 ms, and after the fault is removed, the SC’s recovery time is set as 0.5 s, so as to match up the auto-reclosing operation.

In view of the modeling of the DG3, it includes the controller, LCL filter, three-phase half-bridge voltage converter, chopper and superconducting magnet, and its main structure can be according to (Zhu et al. 2012). It should be noted that, the DG3 will be served as a master-control DG, which is potentially used to stabilize the micro-grid system’s voltage and frequency under the islanded condition.

When the micro-grid is under normal state, the DGs’ overall active power will be controlled as 300 kW (*P*_DG1_ + *P*_DG2_ + *P*_DG3_ = 300 kW). That is to say, the micro-grid’s power shortage with the capacity value of 300 kW will be supported by the main network. Furthermore, the simulation conditions of the external fault are set as that, a three-phase ground fault happens at *t* = 1 s; the fault resistance is 1 Ω; duration of the fault is 0.2 s.

**Fig. 11:**
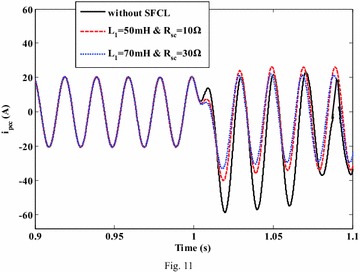
Characteristic of the fault current from the micro-grid side to the PCC point under the external fault (with and without the SFCL)

For the SFCL, different current-limiting parameters are also taken into account, and Fig. 11 shows the fault current from the micro-grid to the PCC (taking the A-phase for example). Since the electromagnetic switch can execute a rapid and reliable interrupting operation after the fault, the SFCL is able to suppress the fault current quickly and responsibly. It is observed that, the current-limiting effects will become more obvious along with the increase of the SFCL’s design parameters, but as the fault current is mainly contributed by the DG units, the maximum amplitude of the fault current will generally not reach to a very high level.

**Fig. 12:**
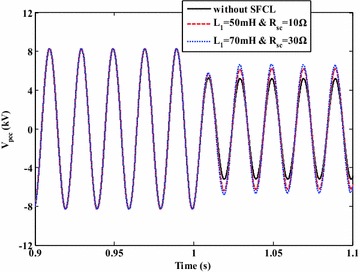
PCC voltage characteristic of the micro-grid system under the external fault (with and without the SFCL)

In view of the conditions with and without the SFCL, Fig. 12 shows the characteristic of the PCC voltage under the external fault (A-phase). From this figure, the PCC voltage will be down to 53 % of the nominal level in the case of without SFCL. When the SFCL is installed and plays the role, the PCC voltage can be improved to 82 % of the nominal level, and it is conducive to enhance the fault ride through capabilities of the IIDG units.

**Fig. 13:**
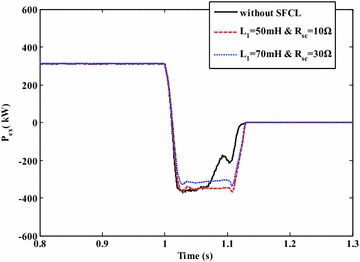
Exchange active power of the micro-grid system at the PCC under the external fault (with and without the SFCL)

**Fig. 14:**
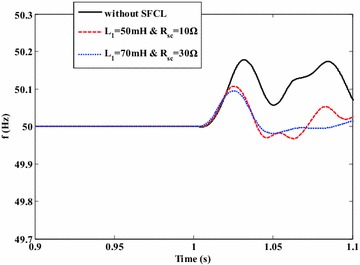
Frequency fluctuations of the micro-grid system under the external fault (with and without the SFCL)

Figures 13 and 14 show the exchange power and frequency fluctuations of the micro-grid system under the external fault. Before the short-circuit fault is cleared by the relay protection at *t* = 1.14 s, the voltage drop in the PCC may deeply affect the power exchange between the main network and the micro-grid. Under this fault, the exchange power’s direction is reverse, and the micro-grid will provide energy to the main network. Owing to the use of the SFCL, the fluctuating margin of the exchange power can be reduced to a certain extent. Moreover, the micro-grid frequency’s fluctuating amplitude can reach to 0.18 Hz in the case of without SFCL, and it may be suppressed within the level of 0.1 Hz when the SFCL is employed.

Note that, different current-limiting parameters of the SFCL are adopted here, and the main reason is to assess how the change of the current-limiting parameters can affect the SFCL’s performance. For the optimization of those parameters, detailed works will be done in the next articles.

## Conclusions

In this paper, the conceptual design of a high-speed switch with electromagnetic repulsion mechanism is performed for a modified flux-coupling-type SFCL, and also the application of the SFCL in a micro-grid with multiple renewable energy sources are assessed. According to theory analysis and parameter design, the electromagnetic switch’s response time can be less than 0.5 ms, and it can meet the requirements of the SFCL for high-speed control. Furthermore, based on the performance simulations of the SFCL equipped with this high-speed switch, the SFCL is able to quickly and availably suppress the fault current contributed by the DG units in the micro-grid system, and meanwhile improve the voltage sag and reduce the frequency fluctuations. As a result, the suggested SFCL’s service practicability can be well confirmed.

In the near future, a small-scale lab prototype of the SFCL will be made, and the current-limiting experiment will be done. Related experiment results will be reported in later articles.
